# Incidence, trends, and outcomes of infection sites among hospitalizations of sepsis: A nationwide study

**DOI:** 10.1371/journal.pone.0227752

**Published:** 2020-01-13

**Authors:** Eric H. Chou, Shaynna Mann, Tzu-Chun Hsu, Wan-Ting Hsu, Carolyn Chia-Yu Liu, Toral Bhakta, Dahlia M. Hassani, Chien-Chang Lee

**Affiliations:** 1 Department of Emergency Medicine, John Peter Smith Hospital, Fort Worth, Texas, United States of America; 2 Department of Emergency Medicine, Baylor Scott and White All Saints Medical Center, Fort Worth, Texas, United States of America; 3 Department of Emergency Medicine, National Taiwan University Hospital and National Taiwan University College of Medicine, Taipei, Taiwan; 4 Department of Epidemiology, Harvard T. H. Chan School of Public Health, Boston, Massachusetts, United States of America; 5 McTimoney College of Chiropractic, School of Health, BPP University, Abingdon, Oxfordshire, United Kingdom; University of Pittsburgh, UNITED STATES

## Abstract

**Purpose:**

To determine the trends of infection sites and outcome of sepsis using a national population-based database.

**Materials and methods:**

Using the Nationwide Inpatient Sample database of the US, adult sepsis hospitalizations and infection sites were identified using a validated approach that selects admissions with explicit ICD-9-CM codes for sepsis and diagnosis/procedure codes for acute organ dysfunctions. The primary outcome was the trend of incidence and in-hospital mortality of specific infection sites in sepsis patients. The secondary outcome was the impact of specific infection sites on in-hospital mortality.

**Results:**

During the 9-year period, we identified 7,860,687 admissions of adult sepsis. Genitourinary tract infection (36.7%), lower respiratory tract infection (36.6%), and systemic fungal infection (9.2%) were the leading three sites of infection in patients with sepsis. Intra-abdominal infection (30.7%), lower respiratory tract infection (27.7%), and biliary tract infection (25.5%) were associated with highest mortality rate. The incidences of all sites of infections were trending upward. Musculoskeletal infection (annual increase: 34.2%) and skin and skin structure infection (annual increase: 23.0%) had the steepest increase. Mortality from all sites of infection has decreased significantly (trend p<0.001). Skin and skin structure infection had the fastest declining rate (annual decrease: 5.5%) followed by primary bacteremia (annual decrease: 5.3%) and catheter related bloodstream infection (annual decrease: 4.8%).

**Conclusions:**

The anatomic site of infection does have a differential impact on the mortality of septic patients. Intra-abdominal infection, lower respiratory tract infection, and biliary tract infection are associated with higher mortality in septic patients.

## Introduction

Being one of the most expensive conditions to treat and a leading cause of death, sepsis has become a major health problem [1, 2]. The incidence of sepsis has been steadily increasing in the past decade, and one recent study estimated an increase in sepsis admissions from 143,000 in 2000 to 343,000 in 2007 [3]. Sepsis was ranked in the top four most costly conditions, costing an aggregate of $20,298,000 million yearly, in US hospitals between all four payer groups (Medicare, Medicaid, private insurers, and uninsured) [4]. This burden on the healthcare system has led to researchers attempting to redefine sepsis and understand its pathophysiologic basis [5, 6]. A recent taskforce led by the Society of Critical Care Medicine and the European Society of Intensive Care Medicine convened and redefined sepsis as life threatening organ dysfunction caused by a dysregulated host response to infection [7]. Current knowledge suggests that mortality in sepsis is related to an overwhelming host immune response to invading pathogens infecting a specific anatomic site, and in current practice the suspected site of infection dictates treatment decisions that impact patient outcome [8]. Therefore, it’s probable that the anatomic infection site may have a significant impact on sepsis mortality. However, there has been a paucity of studies with inconsistent results addressing the various infectious sites effects on mortality, and no reports on the temporal trends of infectious sites and their outcomes [9–12]. Another aspect that could be influenced by studying current trends of infectious sites and their outcome could be researching specific preventative measures tailored towards the most common or highest risk infectious site. Current interventions to prevent certain anatomic site infections are in place such are vaccination against pneumococcal pneumonia or ventilator and line associated bundles [13, 14]. Thus, a study directed towards investigating these issues is important for intensive care resource allocation, public health prevention, and helping prioritize future research.

The primary aim of this study was to delineate the change in the incidence and in-hospital mortality of specific infection sites in sepsis patients over time. The secondary aim was to investigate the effect of anatomic infection site on the in-hospital mortality of sepsis patients.

## Methods

### Data sources

This study was conducted using 2006–2014 data from the Nationwide Inpatient Sample (NIS), part of the Healthcare Cost and Utilization Project, a federal-state-industry partnership sponsored by the Agency for Healthcare Research and Quality (AHRQ). The NIS is the largest all-payer inpatient database in the US, which is a 20% stratified sample of all US community hospitals as defined by the American Hospital Association: nonfederal, short-term, general, and specialty hospitals whose facilities are open to the public. By weighting the patient-level discharge data, it estimates more than 35 million hospitalizations nationally. The database includes clinical variables on all diagnoses and procedures occurring during each hospital admissions. Since the NIS database contains de-identified information regarding each hospitalization, the need for informed consent was waived [15].

### Case selection and definitions

Sepsis hospitalizations were identified using a validated approach that selects admissions with relevant International Classification of Diseases, Ninth Revision, Clinical Modification (ICD-9-CM) diagnosis/procedure codes. Conforming to Sepsis-3 definition, sepsis is defined as life-threatening organ dysfunction caused by a dysregulated host response to infection. The coding system proposed and validated previously by Martin GS et al. is a more conservative estimates that showed a parallel trend with the electronic health record (EHR) estimates [16, 17]. Therefore, we used the Martin’s criteria to identify patients with sepsis in this study. (S2 Table) Sensitivity analysis using Angus criteria was performed to corroborate the results. Operationally, we identified cases with sepsis by selecting all cases with explicit ICD-9-CM codes for sepsis or systemic fungal infection (038 septicemia, 020.0 septicemic, 790.7 bacteremia, 117.9 disseminated fungal infection, 112.5 disseminated candida infection, or 112.81 (disseminated fungal endocarditis) and a diagnosis of acute organ dysfunction. Site of infection was categorized as lower respiratory tract infection, genitourinary tract infection, skin and skin structure infection, catheter related bloodstream infection, intra-abdominal infection, systemic fungal infection, primary bacteremia, musculoskeletal infection, and biliary tract infection (S3 Table). Acute organs/systems dysfunction used for this study was: cardiovascular, respiratory, central nervous system, hematologic, hepatic, renal and metabolic system dysfunction. Shock was included as a form of cardiovascular dysfunction. For patient with multiple diagnoses, only primary and secondary diagnoses were recorded. We used Elixhauser comorbidity Index as our comorbidity index. The following information was collected for analysis: demographic, presence of pre-existing comorbidity, and outcome.

### Outcome measures

The primary outcome was the trend of incidence and in-hospital mortality of specific infection sites in sepsis patients. The secondary outcome was the impact of specific infection sites on in-hospital mortality.

### Statistical analyses

Data management and statistical analyses were conducted using SAS (SAS Inc, Cary, NC) and SAS-callable SUDAAN software (version 9.4, RTI International, Research Triangle, NC) to account for the stratified sampling design used to collect the hospital discharge data. The frequency of hospitalizations for sepsis with specific type of infection was estimated following recommendations from the AHRQ. By using survey-specific statements, SURVEYMEANS in SAS program, we weighted the patient-level discharge data using the weights provided in the NIS database. Continuous variables with normal distribution were presented as mean with standard error (SE), and non-normal variables were reported as median with interquartile range (IQR). Categorical variables were reported as percentage (%). We calculated the overall and average annual percent change in the hospitalization and mortality of sepsis and specific site of infection between 2006 and 2014. To examine the significance of trends of incidence and mortality, we performed linear regression analysis. To evaluate the impact of individual site of infection on the survival of sepsis patients, we fit a multivariable logistic regression model adjusting for age, sex, and comorbidity measures. We used the entire study period (2006 through 2014) for this regression analysis to ensure adequate power to make reliable estimates of risk. Because the mortality rate for patients with sepsis is higher than 10% in this analysis, the rare disease assumption does not hold. As a result, risk ratios cannot be estimated by odds ratios. We used the formula proposed by Zhang and Yu to approximate the relative risk [18]. Two-sided P <0.01 was considered statistically significant for all analyses.

## Results

**Fig 1:**
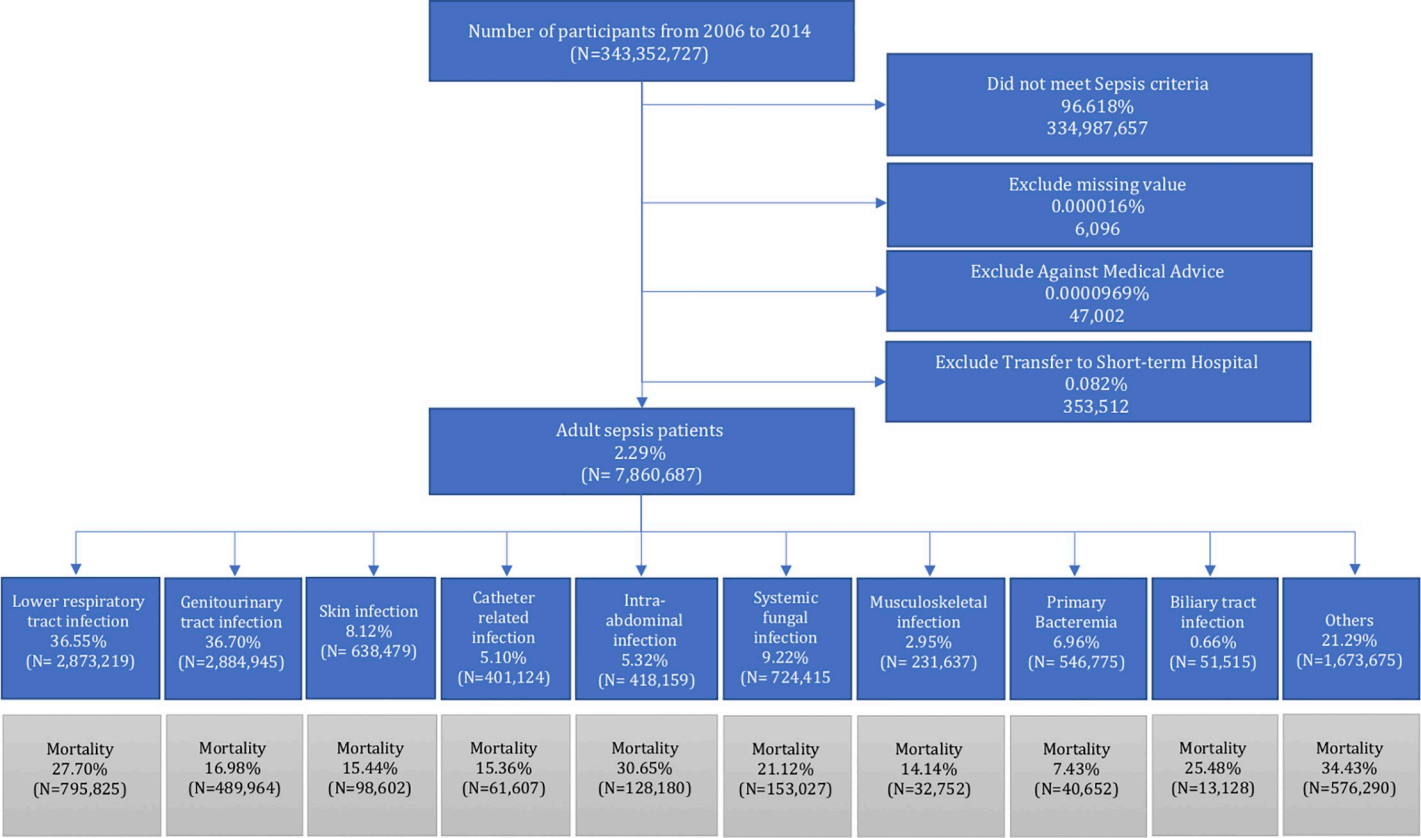
Flowchart of patients in this study.

During the 9 year period between 2006 and 2014, we identified 7,860,687 admissions of adult sepsis. Fig 1 shows the cohort assembling process, total number of each site of infection, corresponding mortality rate, and total number of deaths for each site of infection. Genitourinary tract infection, lower respiratory tract infection and systemic fungal infection were the leading three sites of infection in patients with sepsis, accounting for 36.70%, 36.55% and 9.22% of all sites of infection, respectively. Intra-abdominal infection, lower respiratory tract infection, and biliary tract infection were associated with poor outcome, with a mortality rate of 30.65%, 27.70%, and 25.48%, respectively. Primary bacteremia, musculoskeletal infection, and catheter-related bloodstream infection, however, were associated with better outcome, with a mortality rate of 7.43%, 14.14%, and 15.36%, respectively. Taking the incidence and mortality rate together, lower respiratory tract infection was the leading cause of mortality (weighted death number = 795,825), followed by genitourinary tract infection (weighted death number = 489,964) and systemic fungal infection (weighted death number = 153,027). Table 1 shows the characteristics and sites of infection in the three sub-periods. There are more male patients than female in all subperiods. The mean age of sepsis patients was comparable over the subperiods. The incidence of comorbidities in patients with sepsis increased over the three sub-periods.

**Table 1:** Characteristics of study cohort, stratified by three periods between 2006 and 2014.

Characteristic	2006–2008 n = 1,957,110	2009–2011 n = 2,695,151	2012–2014 n = 3,208,425
Age,yrs	68.22±0.18	67.96±0.17	67.61±0.06
Male sex, %	985407(50.35%)	1366596(50.71%)	1635850(50.99%)
Comorbidity			
Combined comorbidity score	13.47±0.09	14.68±0.1	15.02±0.03
Hypertension	762695(38.97%)	1405676(52.16%)	1869545(58.27%)
Congestive heart failure	482731(24.67%)	654185(24.27%)	795325(24.79%)
Chronic pulmonary disease	444383(22.71%)	657182(24.38%)	848340(26.44%)
Chronic renal failure	488108(24.94%)	746443(27.7%)	928730(28.95%)
Uncomplicated diabetes	329425(16.83%)	628498(23.32%)	818090(25.5%)
Coagulopathy	347156(17.74%)	543134(20.15%)	677745(21.12%)
Neurological disorders	226111(11.55%)	390633(14.49%)	509680(15.89%)
Weight loss	294112(15.03%)	575848(21.37%)	658120(20.51%)
Valvular heart disease	113762(5.81%)	164232(6.09%)	228370(7.12%)
Diabetes with complications	114315(5.84%)	213520(7.92%)	299655(9.34%)
Depression	83354(4.26%)	240851(8.94%)	351695(10.96%)
Peripheral vascular disease	104608(5.35%)	243010(9.02%)	323020(10.07%)
Chronic liver disease	94970(4.85%)	157011(5.83%)	219140(6.83%)
Obesity	68749(3.51%)	262865(9.75%)	451700(14.08%)
Alcohol abuse	75366(3.85%)	120961(4.49%)	169145(5.27%)
Metastatic cancer	101816(5.2%)	137989(5.12%)	166030(5.17%)
Paralysis	96977(4.96%)	184049(6.83%)	222930(6.95%)
Psychoses	61897(3.16%)	134534(4.99%)	185210(5.77%)
Solid tumor	66646(3.41%)	104102(3.86%)	131915(4.11%)
Rheumatic disease	42974(2.2%)	87818(3.26%)	120520(3.76%)
Drug abuse	38803(1.98%)	67415(2.5%)	115210(3.59%)
Lymphoma	42402(2.17%)	57161(2.12%)	66465(2.07%)
AIDS	27871(1.42%)	31230(1.16%)	30165(0.94%)
Sites of infection			
Lower respiratory tract infection	700727(35.8%)	1000502(37.12%)	1171990(36.53%)
Genitourinary tract infection	689089(35.21%)	1010380(37.49%)	1185475(36.95%)
Skin and skin structure infection	133718(6.83%)	218931(8.12%)	285830(8.91%)
Catheter related bloodstream infection	144494(7.38%)	126390(4.69%)	130240(4.06%)
Intra-abdominal infection	95446(4.88%)	147403(5.47%)	175310(5.46%)
Biliary tract infection	11312(0.58%)	18168(0.67%)	22035(0.69%)
Systemic fungal infection	155501(7.95%)	283583(10.52%)	285330(8.89%)
Primary bacteremia	161846(8.27%)	201650(7.48%)	183280(5.71%)
Musculoskeletal infection	42911(2.19%)	83271(3.09%)	105455(3.29%)

n = total episodes of sepsis hospitalization in the subperiod; values are n, mean ± SE, or n (%)

**Fig 2:**
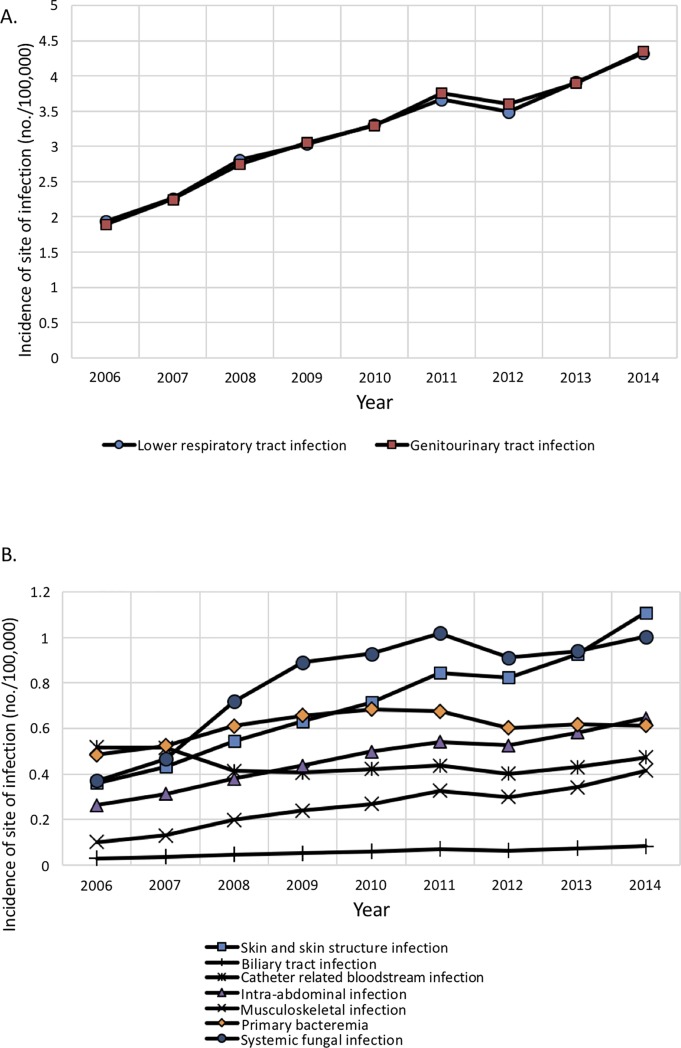
Changes in number of sepsis hospitalizations by specific infection sites among patients with sepsis, from 2006 to 2014. (A) High to moderate number of hospitalizations, (B) low number of hospitalizations.

Fig 2 paired with Table 2 shows the changes in population incidence of specific site of infection in patients with sepsis. The incidence of all sites of infections were trending upward. Musculoskeletal infection, skin and skin structure infection and biliary tract infection had the steepest increase, with an annual increase rate of 34.22%, 23.02% and 20.07%, respectively. On the contrary, Catheter related bloodstream infection and primary bacteremia had a decrease or slow increase, with an annual change rate of -0.97% and 2.89%, respectively. Other sites of infection had an annual increase rate between 13.67% and 18.94%. The aforementioned temporal changes in incidence were all significant (Trend p value <0.001).

**Table 2:** Weighted number of sepsis hospitalizations by specific infection site among patients with sepsis. The annual incidence is presented by events per 100,000 hospitalizations.

	2006	2010	2014	Annual change, %
Lower respiratory tract infection	1.94	3.31	4.32	13.67%
Genitourinary tract infection	1.89	3.29	4.35	14.43%
Intra-abdominal infection	0.26	0.50	0.65	16.15%
Skin and skin structure infection	0.36	0.72	1.11	23.02%
Musculoskeletal infection	0.10	0.27	0.41	34.22%
Primary bacteremia	0.49	0.68	0.61	2.89%
Catheter related bloodstream infection	0.52	0.42	0.47	-0.97%
Systemic fungal infection	0.37	0.93	1.00	18.94%
Biliary tract infection	0.03	0.06	0.08	20.07%

**Fig 3:**
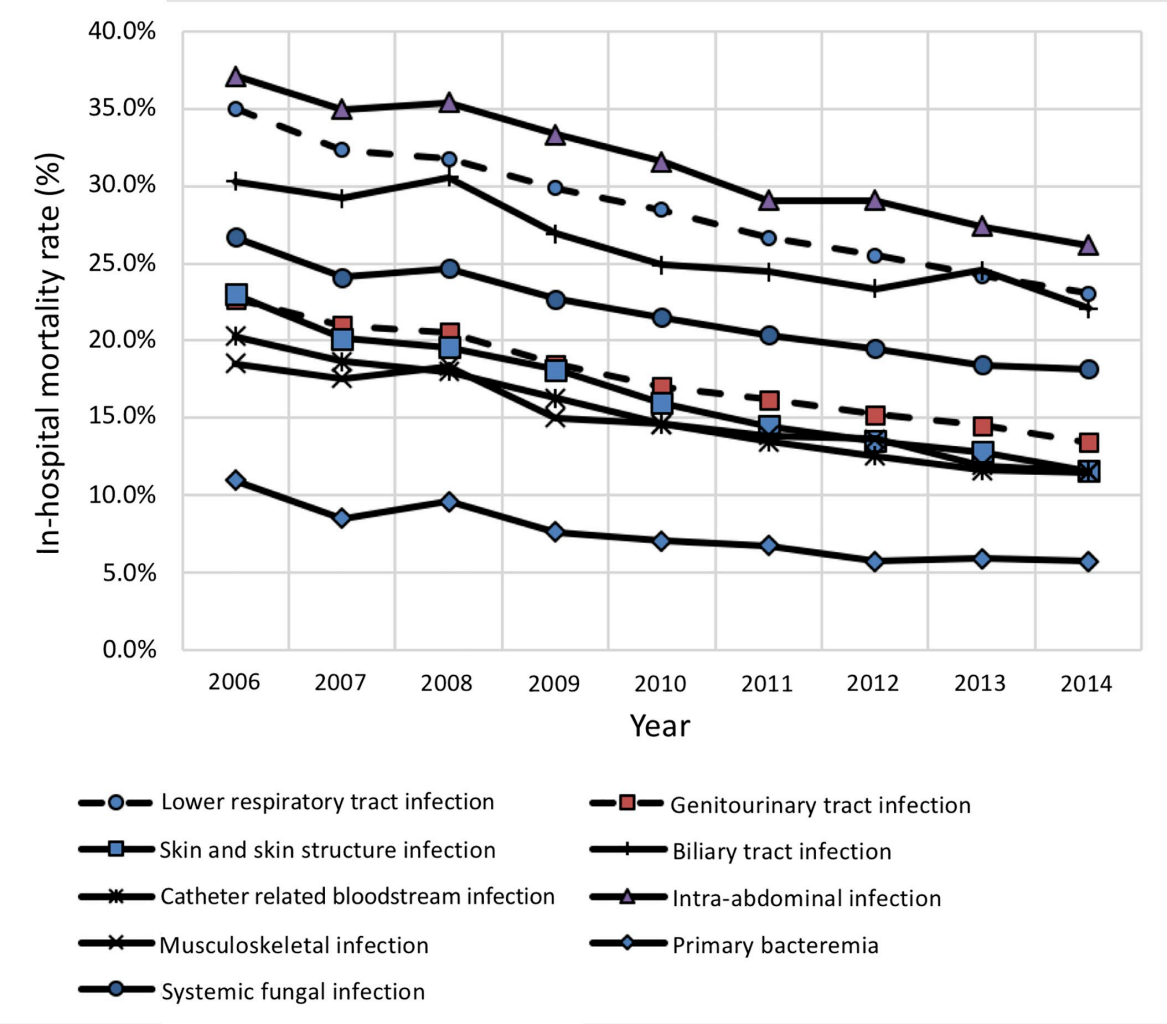
Temporal trend of mortality rate for specific site of infections among patients with sepsis.

Fig 3 paired with Table 3 shows the temporal trends of mortality rate for each infection site in patients with sepsis. Mortality from all sites of infection has decreased significantly in the study period (trend p<0.001). Skin and skin structure had the fastest declining rate (annual decrease: 5.51%) followed by primary bacteremia (annual decrease: 5.32%) and catheter related bloodstream infection (annual decrease: 4.82%).

**Table 3:** In-hospital mortality rate and annual change in rate for specific infection site among patients with sepsis.

	2006	2010	2014	Annual change, %
Lower respiratory tract infection	34.99%	28.46%	23.07%	-3.79%
Genitourinary tract infection	22.70%	17.05%	13.41%	-4.55%
Intra-abdominal infection	37.10%	31.56%	26.18%	-3.27%
Skin and skin structure infection	23.01%	15.99%	11.60%	-5.51%
Musculoskeletal infection	18.55%	14.60%	11.54%	-4.20%
Primary bacteremia	10.98%	7.04%	5.72%	-5.32%
Catheter related bloodstream infection	20.28%	14.63%	11.49%	-4.82%
Systemic fungal infection	26.71%	21.49%	18.18%	-3.55%
Biliary tract infection	30.30%	24.91%	22.08%	-3.01%

**Fig 4:**
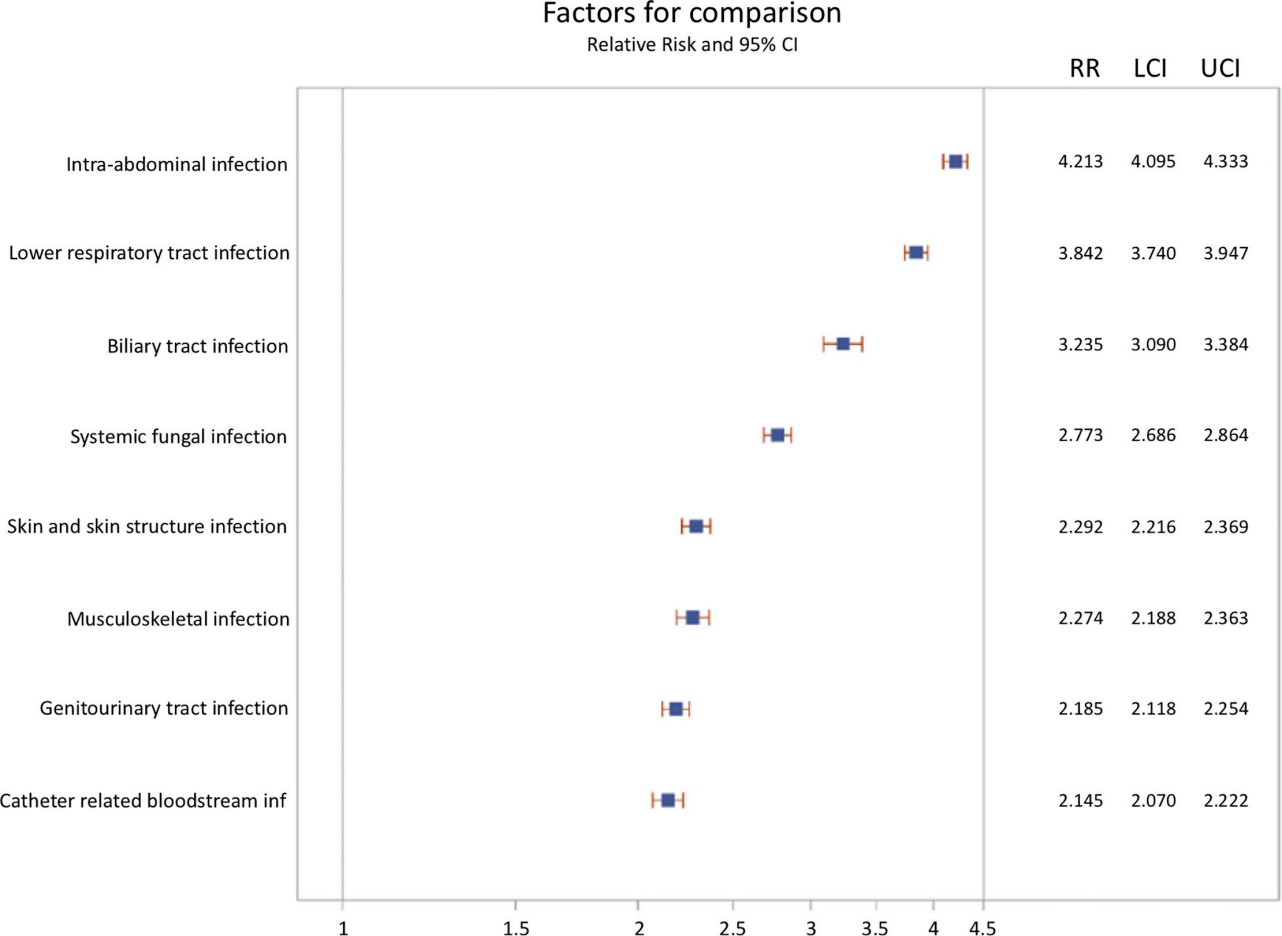
Survival impact of individual infection site in relation to primary bacteremia. The risk estimates were adjusted for all covariates listed in supporting S1 Table. RR refers to the relative risk. LCL and UCL refer to lower and upper confidence limits, respectively.

Fig 4 shows the adjusted relative risk with 95% confidence intervals of infection site on the outcome of sepsis. Using primary bacteremia as reference, sepsis patients with intra-abdominal infection had the highest mortality (RR:4.21), followed by lower respiratory tract infection (RR: 3.84), biliary tract infection (RR: 3.24), systemic fungal infection (RR: 2.77), skin and skin structure infection (RR: 2.29), musculoskeletal infection (RR: 2.27), genitourinary traction infection (RR:2.19), or catheter related bloodstream infection (RR:2.15). Sensitivity analysis with Angus criteria showed similar trend of sepsis as our main results (S4–S7 Tables and S1–S3 Figs)

## Discussion

Based on our study, there has been an increasing trend in the incidence of hospitalizations from sepsis with the greatest number of hospitalizations from lower respiratory tract infections and the least from biliary tract infections. There was also a trend of decreasing mortality from sepsis. Zahar et al suggested that neither site of infection nor presence of bacteremia associated with mortality [9]. However, that study was a single center study with limited sample size. Our study used national database to expand sample size and increase statistic power. Our study showed that, independent of predisposing factors, the site of infection is associated with in hospital mortality in patients with sepsis. Hospital mortality was highest for patients with intra-abdominal infection and lowest for primary bacteremia. This study is the first large national cohort study to investigate a relationship between site of infection and mortality. A few related studies have been performed using smaller sample sizes or different sepsis definitions. They found that either urosepsis or skin infections have a more favorable prognosis while pneumonia or intra-abdominal infection have worse prognosis. Multiple prior studies are consistent with our finding of the trend of increasing sepsis incidence with decreasing mortality [1–3, 19]. This trend is presumably reflecting ongoing efforts to improve sepsis awareness, treatment, documentation, and coding. For example, the surviving sepsis campaign started at the beginning of this study could account for early sepsis recognition and decreasing mortality with early antibiotic administration and three-hour bundle therapy [20–22].

The infection site with the highest incidence in this study was lower respiratory tract infections. Currently preventative strategies mainly aimed at *streptococcus pneumoniae*, which is the common pathogen of pneumonia. After the introduction of pneumococcal vaccinations to both pediatric and adult populations, incidence of pneumococcal pneumonia has decreased [23], A similar strategy of developing further vaccinations or improving upon current vaccinations against other pathogens could minimize predisposition to bacterial pneumonia.

Our study found that sites (intra-abdominal, respiratory, and biliary infections) that have the potential to develop a high burden of organisms resulting in a large downstream pro-inflammatory state caused the highest mortality [8, 24, 25]. In contrast, those infections with multiple protective barriers (such as skin, and musculoskeletal infections) had lower mortality rates [26]. This knowledge could be used to refine prognostication in sepsis helping to select patient populations that may benefit from novel treatments or that require higher levels of monitoring [27–29]. For example, it has been postulated that immunomodulatory agents failed to improve outcomes in septic patients in clinical trials because of enrolment of patients who have lower/intermediate risks or death [30]. Focusing treatments like these on higher mortality sites of infection could have an impact on these infectious sites. The differing mortalities in sites of infection could also be considered in choosing when antibiotic de-escalation is appropriate. Those patient with lower risk of mortality might benefit from early de-escalation of the antibiotics. Helping to reduce antimicrobial resistance, and adverse drug reactions [31, 32].

There were several limitations of this study. First, identifying sepsis using ICD-9 CM codes algorithm may not be as precise as screening EHR with clinical criteria because clinicians and hospital coders may vary widely in their knowledge and application of sepsis definitions [33]. However, the estimation of sepsis trend using EHR from several hospitals demands a lot of resources. In addition, different hospitals contributed data of different years with different case mix lowers the generalizability of the estimation. Previous studies showed sepsis estimated from Martin’s algorithm is a conservative and reasonable proxy to the estimates from EHR, therefore we adopted Martin’s implementation for this study [17, 34]. Second, although we adjusted for multiple factors that could influence hospital mortality, there may be other confounding factors that we did not account for and measure. Third, we assumed organ dysfunction to be a downstream effect of serious infection, and thus did not adjust for organ dysfunction in the regression model avoiding intermediate bias. Fourth, by using in-hospital mortality as our endpoint overall mortality of specific infections may have been underestimated if the events occurred outside of the hospital. Moreover, our administrative data is unable to establish a firm temporal relationship between sepsis and the onset of organ dysfunction. Meanwhile, due to the insufficient information from the database, we cannot identify patients’ socioeconomical status, community-acquired or nosocomial, medical or surgical hospitalization. Also, we didn’t perform the control group analysis due to insufficient data. Further study involving more detailed in-patient data is required. In order to increase the comparability of our study, we used Angus implementation for the recognition of infection sites. The Angus implementation was originally invented for the identification of severe sepsis with an ICD-9 based criteria, which is by far one of the most widely used implementations [9, 34–38]. Using the same criteria as other studies could increase the comparability and provide opportunity for future meta-analysis. However, some of the specific diagnoses may not be included. Lastly, our results may not be generalizable to other parts of the world because this study was conducted in American hospitals. Further studies would be needed to address these limitations and provide explanation to this trend.

## Conclusion

There is a significant difference in the trend of incidence and outcome of sepsis from different anatomic sites of infection. Clinicians should be aware of different anatomic sites of infection could cause higher mortality in septic patients such as intra-abdominal infection, lower respiratory tract infection, and biliary tract infection.

## Supporting information

S1 Fig Sensitivity Test—Changes in number of sepsis hospitalizations by specific infection sites among patients with sepsis, from 2006 to 2014. (A) High to moderate number of hospitalizations, (B) low number of hospitalizations.
(TIFF)

S2 Fig Sensitivity Test—Temporal trend of mortality rate for specific source of infections among patients with sepsis. (TIFF)

S3 Fig Sensitivity Test—Survival impact of individual infection site in relation to primary bacteremia. The risk estimates were adjusted for all covariates listed in Supporting S7 Table. RR refers to the relative risk. LCL and UCL refer to lower and upper confidence limits, respectively.
(TIFF)

S1 Table Covariates with associated relative risk in the outcome regression model. (PDF)

S2 Table ICD-9 Code associated with organ dysfunction. (PDF)

S3 Table ICD-9-CM codes of site of infections associated with sepsis. (PDF)

S4 Table Sensitivity Test—Characteristics of study cohort, stratified by three periods between 2006 and 2014. (PDF)

S5 Table Sensitivity Test—Number of sepsis hospitalizations by specific infection site among patients with sepsis. The annual incidence is presented by events per 100,000 hospitalizations.
(PDF)

S6 Table Sensitivity Test—In-hospital mortality rate and annual change in rate for specific infection site among patients with sepsis. (PDF)

S7 Table Sensitivity Test—Covariates with associated relative risk in the outcome regression model. (PDF)
