# Supplementary figures and images for: Incidence, trends, and outcomes of infection sites among hospitalizations of sepsis: A nationwide study
Source: PLoS One. 2020 Jan 13;15(1):e0227752. doi: 10.1371/journal.pone.0227752 (PMC6957188; doi:10.1371/journal.pone.0227752)

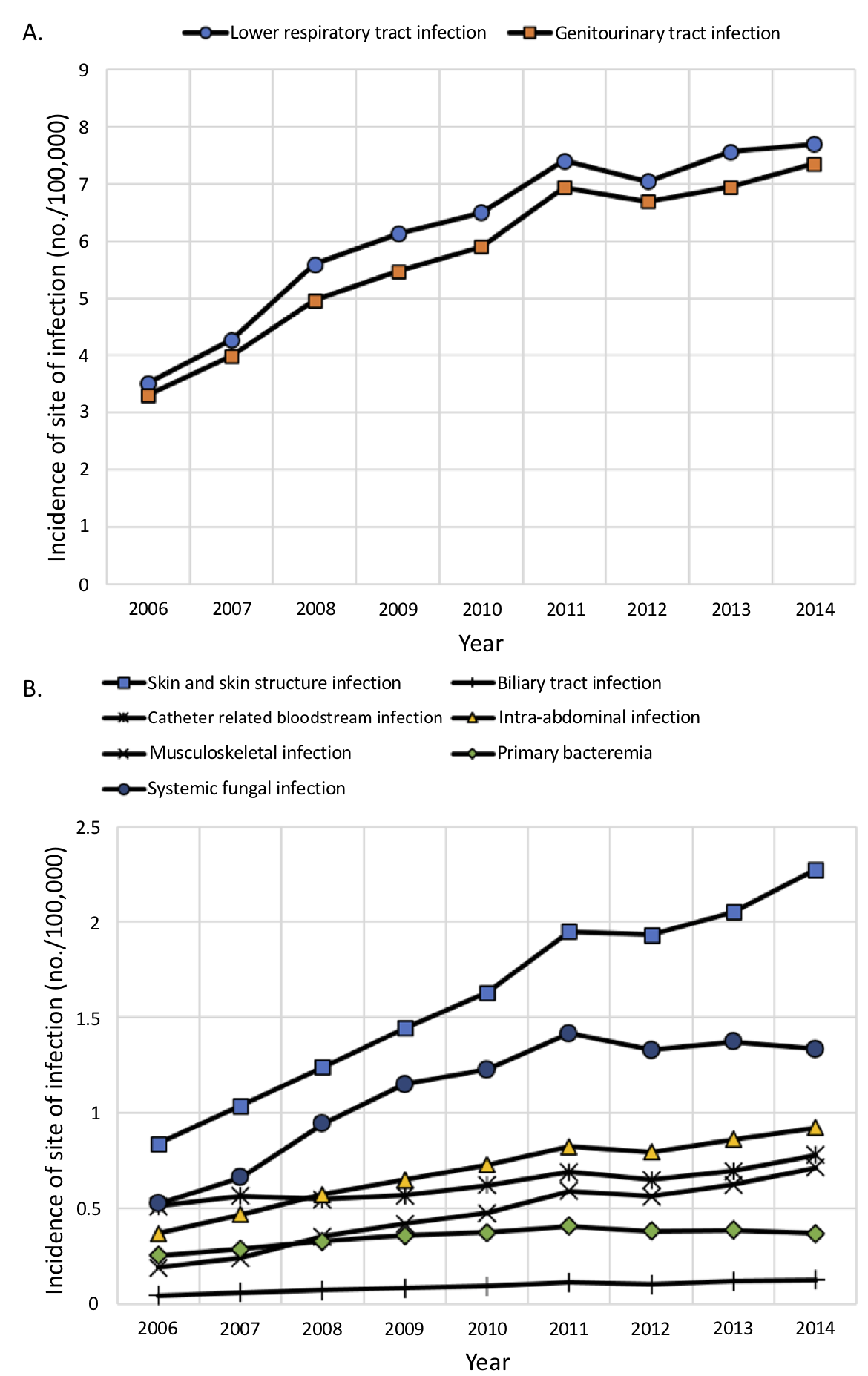

Supplement: S1 Fig — (A) High to moderate number of hospitalizations, (B) low number of hospitalizations. (TIFF) [file pone.0227752.s001.tiff]

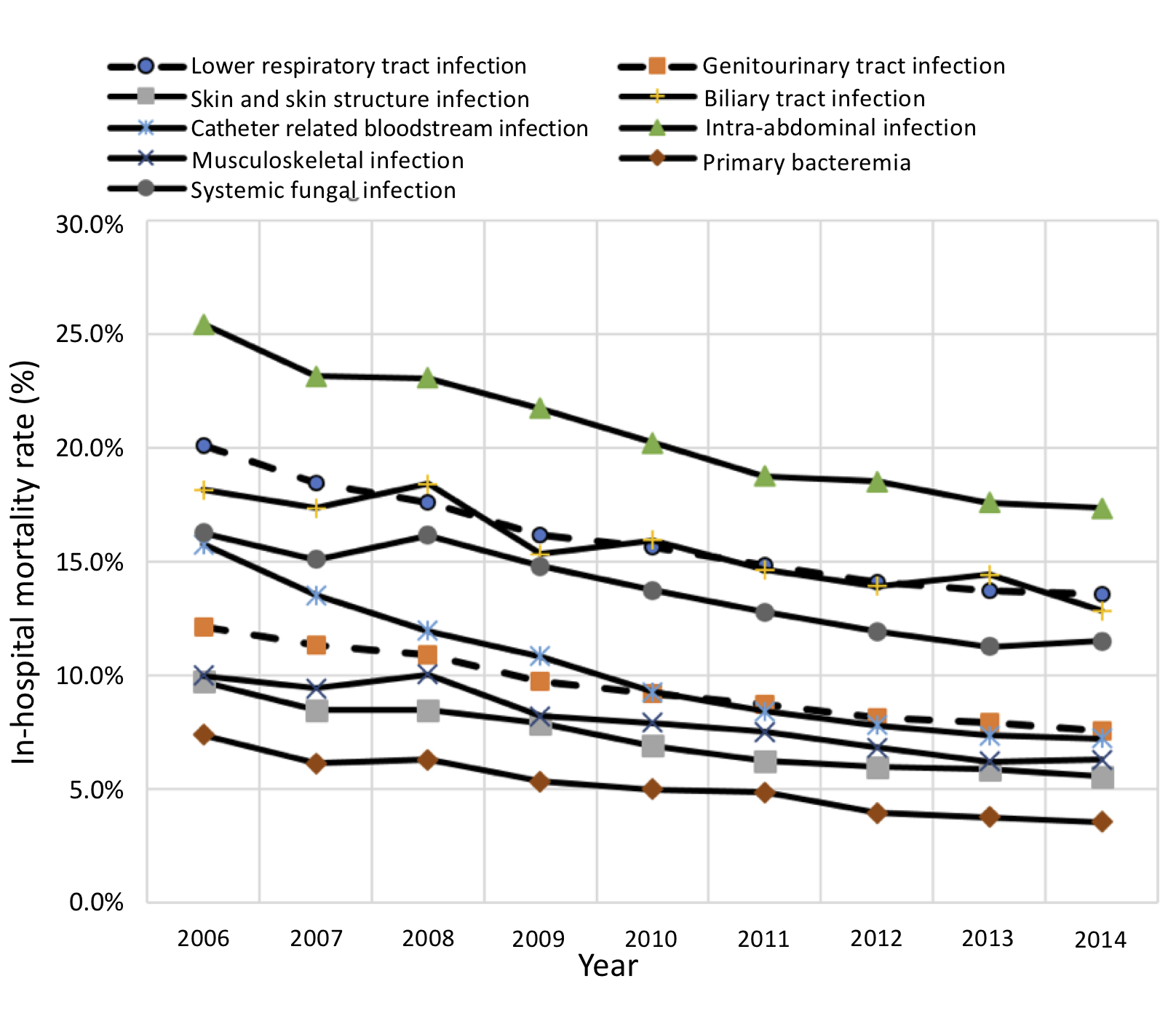

Supplement: S2 Fig — (TIFF) [file pone.0227752.s002.tiff]

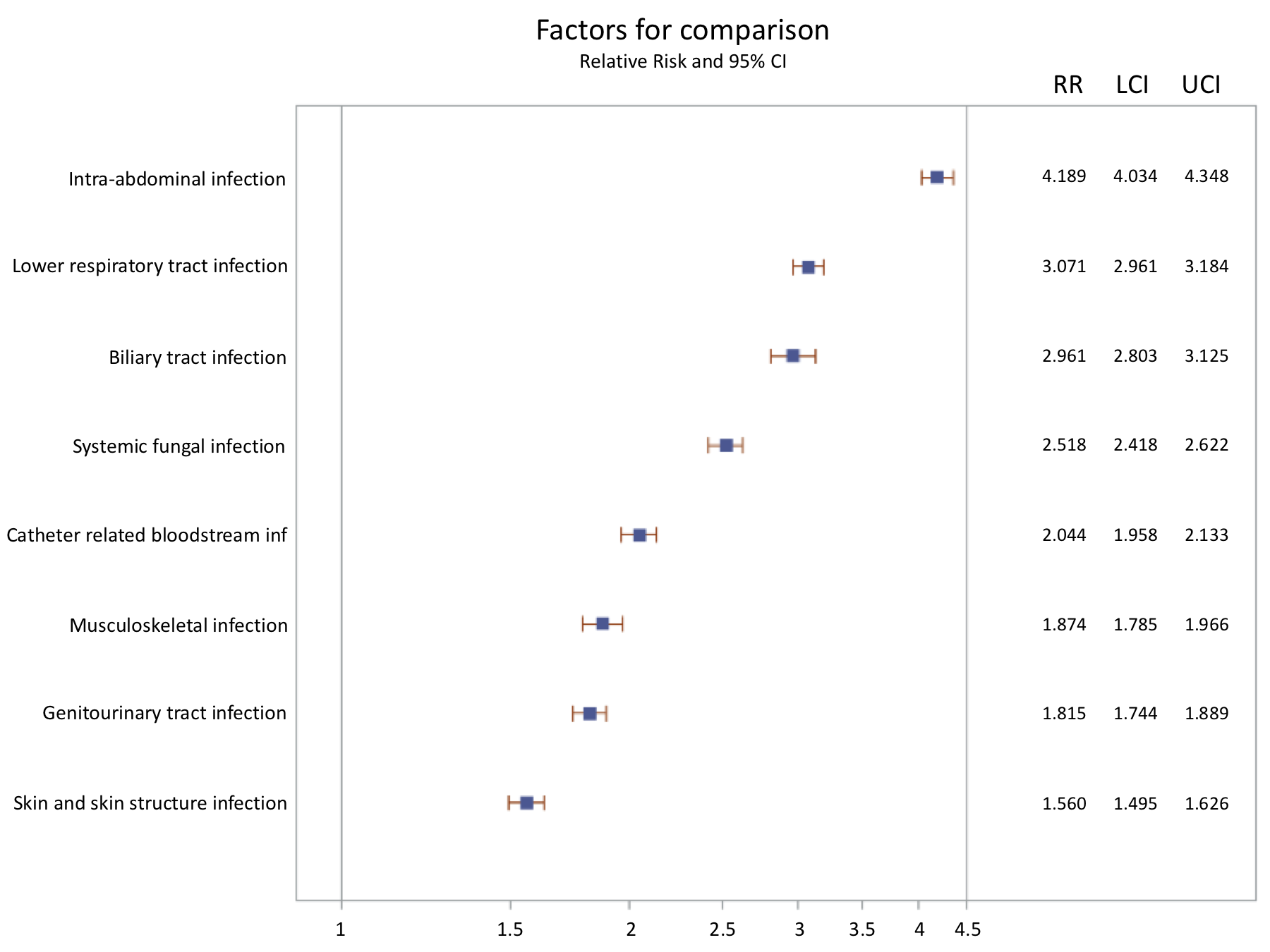

Supplement: S3 Fig — The risk estimates were adjusted for all covariates listed in Supporting S7 Table. RR refers to the relative risk. LCL and UCL refer to lower and upper confidence limits, respectively. (TIFF) [file pone.0227752.s003.tiff]
