# Supplementary material for: Incidence, trends, and outcomes of infection sites among hospitalizations of sepsis: A nationwide study
Source: PLoS One. 2020 Jan 13;15(1):e0227752. doi: 10.1371/journal.pone.0227752 (PMC6957188; doi:10.1371/journal.pone.0227752)
Supplement: S1 Table — (PDF) [file pone.0227752.s004.pdf]

S1 Table. Covariates with associated relative risk in the outcome regression model.

	Relative Risk (95% Confidence Interval)	P-value
Site of infections		
Primary bacteremia	Ref	
Intra-abdominal infection	4.21(4.09,4.33)	<.0001
Lower respiratory tract infection	3.84(3.74,3.95)	<.0001
Biliary tract infection	3.23(3.09,3.38)	<.0001
Systemic fungal infection	2.77(2.69,2.86)	<.0001
Skin and skin structure infection	2.29(2.22,2.37)	<.0001
Musculoskeletal infection	2.27(2.19,2.36)	<.0001
Genitourinary tract infection	2.19(2.12,2.25)	<.0001
Catheter related bloodstream infection	2.14(2.07,2.22)	<.0001
Male gender	0.91(0.9,0.92)	<.0001
Age	1.02(1.02,1.02)	<.0001
Hypertension	0.73(0.72,0.73)	<.0001
Congestive heart failure	1.02(1,1.04)	0.0289
Valvular heart disease	0.9(0.89,0.92)	<.0001
Peripheral vascular disease	1.14(1.12,1.16)	<.0001
Chronic pulmonary disease	0.82(0.8,0.83)	<.0001
Chronic renal failure	1.01(1,1.03)	0.0777
Chronic liver disease	1.57(1.53,1.6)	<.0001
Uncomplicated diabetes	0.85(0.84,0.86)	<.0001
Diabetes with complications	0.73(0.71,0.74)	<.0001
Rheumatic disease	0.89(0.87,0.91)	<.0001
Neurological disorders	0.85(0.83,0.86)	<.0001

Paralysis	0.88(0.86,0.9)	<.0001
Solid tumor	1.15(1.12,1.18)	<.0001
Metastatic cancer	1.67(1.63,1.72)	<.0001
Lymphoma	1.27(1.23,1.31)	<.0001
Acquired immune deficiency syndrome	1.66(1.57,1.76)	<.0001
Coagulopathy	1.17(1.15,1.19)	<.0001
Alcohol abuse	1.11(1.08,1.14)	<.0001
Drug abuse	0.86(0.82,0.89)	<.0001
Psychoses	0.68(0.66,0.69)	<.0001
Depression	0.72(0.7,0.73)	<.0001
Obesity	0.85(0.83,0.86)	<.0001
Weight loss	0.94(0.92,0.96)	<.0001
Combined comorbidity score	1.02(1.02,1.03)	<.0001
Combined comorbidity score Squared	1(1,1)	<.0001
