# Supplementary material for: Incidence, trends, and outcomes of infection sites among hospitalizations of sepsis: A nationwide study
Source: PLoS One. 2020 Jan 13;15(1):e0227752. doi: 10.1371/journal.pone.0227752 (PMC6957188; doi:10.1371/journal.pone.0227752)
Supplement: S2 Table — (PDF) [file pone.0227752.s005.pdf]

S2 Table. ICD-9 Code associated with organ dysfunction

Cardiovascular dysfunction/Shock

785.5 Shock

458 Hypotension

Acute respiratory failure

96.7 Mechanical ventilation

(57001B, 57002B, 57023B, 57029C) Use of ventilator

Central nervous system dysfunction

348.3 Neurologic Encephalopathy

293 Transient organic psychosis

348.1 Anoxic brain damage

Hematologic system dysfunction

287.4 Hematologic Secondary thrombocytopenia

287.5 Thrombocytopenia, unspecified

286.9 Other/unspecified coagulation defect

286.6 Defibrination syndrome

Hepatic system dysfunction

570 Hepatic Acute and subacute necrosis of liver

572.2 Hepatic encephalopathy

572,4 Hepatorenal syndrome

572.8 Other squeal of chronic liver disease

573.4, 573.8 Hepatic infarction

V42.7 Liver replaced by transplant

Renal system dysfunction

584 Acute renal failure

(58014C) Use of CVVH

Metabolic system dysfunction

250.1 Diabetic ketoacidosis

250.2 Hyperosmolar hyperglycemic state
