# Supplementary material for: Incidence, trends, and outcomes of infection sites among hospitalizations of sepsis: A nationwide study
Source: PLoS One. 2020 Jan 13;15(1):e0227752. doi: 10.1371/journal.pone.0227752 (PMC6957188; doi:10.1371/journal.pone.0227752)
Supplement: S3 Table — (PDF) [file pone.0227752.s006.pdf]

S3 Table. ICD-9-CM codes of site of infections associated with sepsis

Lower respiratory tract infection

481, Pneumococcal pneumonia

482, Other bacterial pneumonia

485, Bronchopneumonia with organism not otherwise specified

486, Pneumonia, organism not otherwise specified

491.21, Acute exacerbation of obstructive chronic bronchitis

494, Bronchiectasis

510, Empyema

513, Lung/mediastinum abscess

033, Whooping cough

484, Pneumonia classified in elsewhere

483, Pneumonia by other pathogens

Genitourinary tract infection

590, Kidney infection

597, Urethritis/urethral syndrome

599.0, Urinary tract infection not otherwise specified

601, Prostatic inflammation

098, Gonococcal infections

Intra-abdominal infection

540, Acute appendicitis

541, Appendicitis not otherwise specified

542, Other appendicitis

566, Anal and rectal abscess

567, Peritonitis

569.5, Intestinal abscess

569.83, Perforation of intestine

572.0, Abscess of liver

Skin and skin structure infection

682, Other cellulitis or abscess

683, Acute lymphadenitis

686, Other local skin infection

035, Erysipelas

Musculoskeletal infection

711.0, Pyogenic arthritis

730, Osteomyelitis

Primary bacteremia

790.7, Bacteremia

Catheter related bloodstream infection

996.6, Infection or inflammation of device/graft;

Systemic fungal infection

110, Dermatophytosis

111, Dermatomycosis not otherwise classified or specified

112, Candidiasis

114, Coccidioidomycosis

115, Histoplasmosis

116, Blastomycotic infection

117, Other mycoses

118, Opportunistic mycoses

117.9, Disseminated fungal infection

112.5, Dissemintaed candidal infection

112.81, Disseminated fungal endocarditis

039, Actinomycotic infections

Biliary tract infection

572.1, Portal pyremia

575.0, Acute cholecystitis
