# Supplementary material for: Incidence, trends, and outcomes of infection sites among hospitalizations of sepsis: A nationwide study
Source: PLoS One. 2020 Jan 13;15(1):e0227752. doi: 10.1371/journal.pone.0227752 (PMC6957188; doi:10.1371/journal.pone.0227752)
Supplement: S4 Table — (PDF) [file pone.0227752.s007.pdf]

S4 Table. Sensitivity Test - Characteristics of study cohort, stratified by three periods between 2006 and 2014

Characteristic	2006-2008<br>n=3225623	2009-2011<br>n=4804726	2012-2014<br>n=5603935
Age,yrs	69.83±0.15	69.74±0.15	69.37±0.05
Male sex, %	1532420(47.51%)	2295601(47.78%)	2702610(48.23%)
Comorbidity			
Combined comorbidity score	12.68±0.07	13.36±0.08	13.54±0.02
Hypertension	1528277(47.38%)	2699051(56.17%)	3416565(60.97%)
Congestive heart failure	767325(23.79%)	1120847(23.33%)	1321435(23.58%)
Chronic pulmonary disease	816771(25.32%)	1240755(25.82%)	1498390(26.74%)
Chronic renal failure	810842(25.14%)	1355530(28.21%)	1683150(30.04%)
Uncomplicated diabetes	643062(19.94%)	1190514(24.78%)	1482940(26.46%)
Coagulopathy	642333(19.91%)	1011832(21.06%)	1175830(20.98%)
Neurological disorders	353284(10.95%)	677960(14.11%)	834495(14.89%)
Weight loss	346269(10.73%)	702503(14.62%)	819345(14.62%)
Valvular heart disease	204861(6.35%)	308792(6.43%)	399350(7.13%)
Diabetes with complications	227762(7.06%)	413691(8.61%)	546995(9.76%)
Depression	232269(7.2%)	535787(11.15%)	709875(12.67%)
Peripheral vascular disease	202861(6.29%)	440694(9.17%)	559925(9.99%)
Chronic liver disease	150075(4.65%)	260744(5.43%)	348445(6.22%)
Obesity	162421(5.04%)	495562(10.31%)	793025(14.15%)
Alcohol abuse	111425(3.45%)	193865(4.03%)	256815(4.58%)
Metastatic cancer	130390(4.04%)	198338(4.13%)	235475(4.2%)
Paralysis	131231(4.07%)	238167(4.96%)	283210(5.05%)
Psychoses	124714(3.87%)	251578(5.24%)	326180(5.82%)
Solid tumor	107677(3.34%)	176927(3.68%)	211975(3.78%)

Rheumatic disease	89573(2.78%)	172203(3.58%)	220145(3.93%)
Drug abuse	62169(1.93%)	112375(2.34%)	178240(3.18%)
Lymphoma	60956(1.89%)	88473(1.84%)	103860(1.85%)
AIDS	36783(1.14%)	34564(0.72%)	32915(0.59%)
Site of infection			
Lower respiratory tract infection	1340070(41.54%)	2004510(41.72%)	2231965(39.83%)
Genitourinary tract infection	1226251(38.02%)	1831995(38.13%)	2101040(37.49%)
Skin and skin structure infection	311797(9.67%)	502371(10.46%)	625730(11.17%)
Catheter related bloodstream infection	162568(5.04%)	188154(3.92%)	212500(3.79%)
Intra-abdominal infection	140929(4.37%)	220231(4.58%)	257840(4.6%)
Biliary tract infection	18000(0.56%)	29076(0.61%)	34925(0.62%)
Systemic fungal infection	213130(6.61%)	379555(7.9%)	403925(7.21%)
Primary bacteremia	86870(2.69%)	114229(2.38%)	113745(2.03%)
Musculoskeletal infection	78477(2.43%)	148723(3.1%)	190075(3.39%)

n= total episodes of sepsis hospitalization in the subperiod; values are n, mean ± SE, or n (%)
