# Supplementary material for: Incidence, trends, and outcomes of infection sites among hospitalizations of sepsis: A nationwide study
Source: PLoS One. 2020 Jan 13;15(1):e0227752. doi: 10.1371/journal.pone.0227752 (PMC6957188; doi:10.1371/journal.pone.0227752)
Supplement: S5 Table — The annual incidence is presented by events per 100,000 hospitalizations. (PDF) [file pone.0227752.s008.pdf]

S5 Table. Sensitivity Test - Number of sepsis hospitalizations by specific infection site among patients with sepsis. The annual incidence is presented by events per 100,000 hospitalizations.

	**2006**	**2010**	**2014**	**Annual change, %**
Lower respiratory tract infection	3.52	6.50	7.70	13.17
Genitourinary tract infection	3.31	5.90	7.36	13.56
Intra-abdominal infection	0.37	0.73	0.92	16.73
Skin and skin structure infection	0.84	1.63	2.27	19.01
Musculoskeletal infection	0.19	0.48	0.71	30.42
Primary bacteremia	0.25	0.37	0.37	4.99
Catheter related bloodstream infection	0.51	0.62	0.78	5.79
Systemic fungal infection	0.52	1.23	1.34	17.22
Biliary tract infection	0.05	0.09	0.13	19.85
