# Supplementary material for: Incidence, trends, and outcomes of infection sites among hospitalizations of sepsis: A nationwide study
Source: PLoS One. 2020 Jan 13;15(1):e0227752. doi: 10.1371/journal.pone.0227752 (PMC6957188; doi:10.1371/journal.pone.0227752)
Supplement: S6 Table — (PDF) [file pone.0227752.s009.pdf]

S6 Table. Sensitivity Test - In-hospital mortality rate and annual change in rate for specific infection site among patients with sepsis.

	2006	2010	2014	Annual change, %
Lower respiratory tract infection	20.14%	15.64%	13.60%	-3.61%
Genitourinary tract infection	12.16%	9.19%	7.59%	-4.18%
Intra-abdominal infection	25.44%	20.22%	17.36%	-3.53%
Skin and skin structure infection	9.73%	6.90%	5.55%	-4.77%
Musculoskeletal infection	9.99%	7.92%	6.30%	-4.11%
Primary bacteremia	7.39%	5.00%	3.57%	-5.74%
Catheter related bloodstream infection	15.78%	9.27%	7.23%	-6.02%
Systemic fungal infection	16.27%	13.75%	11.51%	-3.25%
Biliary tract infection	18.16%	15.96%	12.84%	-3.26%
