# Supplementary material for: Incidence, trends, and outcomes of infection sites among hospitalizations of sepsis: A nationwide study
Source: PLoS One. 2020 Jan 13;15(1):e0227752. doi: 10.1371/journal.pone.0227752 (PMC6957188; doi:10.1371/journal.pone.0227752)
Supplement: S7 Table — (PDF) [file pone.0227752.s010.pdf]

S7 Table. Sensitivity Test - Covariates with associated relative risk in the outcome regression model.

	**Relative Risk (95% Confidence Interval)**	**P-value**
Site of infection		
Primary bacteremia	Ref	
Intra-abdominal infection	4.19(4.03,4.35)	<.0001
Lower respiratory tract infection	3.07(2.96,3.18)	<.0001
Biliary tract infection	2.96(2.80,3.13)	<.0001
Systemic fungal infection	2.52(2.42,2.62)	<.0001
Catheter related bloodstream infection	2.04(1.96,2.13)	<.0001
Musculoskeletal infection	1.87(1.78,1.97)	<.0001
Genitourinary tract infection	1.82(1.74,1.89)	<.0001
Skin and skin structure infection	1.56(1.49,1.63)	<.0001
Male gender	0.91(0.90,0.92)	<.0001
Age	1.02(1.02,1.02)	<.0001
Income quartile, U.S. $		
Lowest quartile	Ref	<.0001
2nd quartile	0.93(0.92,0.95)	
3rd quartile	0.92(0.91,0.94)	
Highest quartile	0.96(0.93,0.99)	
Comorbidity		
Hypertension	0.72(0.71,0.72)	<.0001
Congestive heart failure	0.97(0.96,0.99)	0.001
Valvular heart disease	0.90(0.89,0.92)	<.0001
Peripheral vascular disease	0.98(0.97,1.00)	0.0463
Chronic pulmonary disease	0.80(0.79,0.81)	<.0001

Chronic renal failure	0.84(0.83,0.86)	<.0001
Chronic liver disease	1.38(1.35,1.41)	<.0001
Uncomplicated diabetes	0.83(0.82,0.84)	<.0001
Diabetes with complications	0.73(0.71,0.75)	<.0001
Rheumatic disease	0.89(0.87,0.91)	<.0001
Neurological disorders	0.85(0.84,0.87)	<.0001
Paralysis	1.03(1.01,1.06)	0.0044
Solid tumor	1.03(1.01,1.06)	0.0093
Metastatic cancer	1.45(1.41,1.48)	<.0001
Lymphoma	1.19(1.15,1.22)	<.0001
Acquired immune deficiency syndrome	1.43(1.34,1.51)	<.0001
Coagulopathy	0.81(0.79,0.82)	<.0001
Alcohol abuse	1.10(1.07,1.12)	<.0001
Drug abuse	1.06(1.02,1.10)	0.0053
Psychoses	0.75(0.73,0.77)	<.0001
Depression	0.71(0.70,0.73)	<.0001
Obesity	0.92(0.90,0.94)	<.0001
Weight loss	1.08(1.06,1.10)	<.0001
Combined comorbidity score	1.05(1.05,1.05)	<.0001
Combined comorbidity score Squared	1.00(1.00,1.00)	<.0001
