# Supplementary material for: Incidence, trends, and outcomes of infection sites among hospitalizations of sepsis: A nationwide study
Source: PLoS One. 2020 Jan 13;15(1):e0227752. doi: 10.1371/journal.pone.0227752 (PMC6957188; doi:10.1371/journal.pone.0227752)
Supplement: Attachment — Submitted filename: Chou et al.docx [file pone.0227752.s011.docx]

Chou *et al.* have significantly improved the methodology of this study. The interpretation of results is sound with expanded limitations and discussion. I agree with publication of these important results with minor revisions listed below.

Minor revisions:

1) Line 151-152: This sentence is confusing. Please re-word. “Male patients tend to be more likely to sepsis in all subperiods.”
2) Lines 182-189: You use Odds Ratios, but in the methods section lines 133-134 you mention that odds ratios are biased measures given the prevalence of sepsis and therefore do not reliably predict risk. Please clarify.
3) Lines 209-210: “One example being the surviving sepsis campaign being started at the beginning of this study who could account for increased incidence of sepsis”. This is confusing. Please re-word.
4) Lines 214-215: “which is common etiologic agent of pneumonia.” Please fix grammar.
