# Supplementary material for: Incidence, trends, and outcomes of infection sites among hospitalizations of sepsis: A nationwide study
Source: PLoS One. 2020 Jan 13;15(1):e0227752. doi: 10.1371/journal.pone.0227752 (PMC6957188; doi:10.1371/journal.pone.0227752)
Supplement: Attachment — Submitted filename: Response to Reviewers 2nd revision.doc [file pone.0227752.s012.doc]

Ref: PONE-D-19-15923

Title: Incidence, trends, and outcomes of infection sites among hospitalizations of sepsis: a nationwide study
Journal: PLOS ONE

Dear editor and reviewers:

Thank you for your e-mail with the referees’ comments. We have revised the manuscript as advised. The detailed point-by-point responses are as follows. We hope the manuscript is now acceptable to PLOS ONE.

Editor’s Comment:

Thank you for submitting your manuscript to PLOS ONE. After careful consideration, we feel that it has merit but does not fully meet PLOS ONE’s publication criteria as it currently stands. Therefore, we invite you to submit a revised version of the manuscript that addresses the points raised during the review process.

Please make sure to address the additional comments raised by the reviewers. In particular, please justify the use of odds / odds ratios for risk prediction given its known limitations (e.g., Pepe MS, Am J Epidemiol 2004). Furthermore, please include sensitivity analyses using alternative sepsis coding strategy (e.g., 'Angus methodology') in the supplement section.

**Author Reply:**

Thanks for your comment. To evaluate the impact of individual site of infection on the survival of sepsis patients, we fit a multivariable logistic regression model adjusting for age, sex, and comorbidity measures. We used the entire study period for this regression analysis to ensure adequate power to make reliable estimates of risk. Because the mortality rate for patients with sepsis is higher than 10% in this analysis, the rare disease assumption does not hold. As a result, risk ratios cannot be estimated by odds ratios. Therefore, we used the formula proposed by Zhang and Yu to approximate the relative risk. (1) We have corrected our manuscript and figures accordingly.

“As a result, risk ratios cannot be estimated by odds ratios.” (Line 134 in Section of ***Methods***)

“Figure 4 shows the adjusted relative risk with 95% confidence intervals of infection site on the outcome of sepsis. Using primary bacteremia as reference, sepsis patients with intra-abdominal infection had the highest mortality (RR:4.21), followed by lower respiratory tract infection (RR: 3.84), biliary tract infection (RR: 3.24), systemic fungal infection (RR: 2.77), skin and skin structure infection (RR: 2.29), musculoskeletal infection (RR: 2.27), genitourinary traction infection (RR:2.19), or catheter related bloodstream infection (RR:2.15). Sensitivity analysis with Angus criteria showed similar trend of sepsis as our main results (Table S4-7 and Fig S1-3)

Fig 4. Survival impact of individual infection site in relation to primary bacteremia. The risk estimates were adjusted for all covariates listed in supporting table 1. RR refers to the relative risk. LCL and UCL refer to lower and upper confidence limits, respectively.” (Line 182-191 in Section of ***Results***)

“Supporting Figure 3. Sensitivity Test - Survival impact of individual infection site in relation to primary bacteremia. The risk estimates were adjusted for all covariates listed in Supporting table 7. RR refers to the relative risk. LCL and UCL refer to lower and upper confidence limits, respectively.

Supporting Table 1. Covariates with associated relative risk in the outcome regression model.” (Line 371-376 in Section of ***Supporting Information***)

“Supporting Table 7. Sensitivity Test - Covariates with associated relative risk in the outcome regression model.” (Line 386-388 in Section of ***Supporting Information***)

We have included sensitivity analyses using Angus methodology in Table S4-7 and Fig S1-3.

We would appreciate receiving your revised manuscript by Jan 02 2020 11:59PM. When you are ready to submit your revision, log on to <https://www.editorialmanager.com/pone/> and select the 'Submissions Needing Revision' folder to locate your manuscript file.

If you would like to make changes to your financial disclosure, please include your updated statement in your cover letter.

To enhance the reproducibility of your results, we recommend that if applicable you deposit your laboratory protocols in [protocols.io](http://protocols.io/), where a protocol can be assigned its own identifier (DOI) such that it can be cited independently in the future. For instructions see: [http://journals.plos.org/plosone/s/submission-guidelines#loc-laboratory-protocols](http://journals.plos.org/plosone/s/submission-guidelines#loc-laboratory-protocols)

Please include the following items when submitting your revised manuscript:

- A rebuttal letter that responds to each point raised by the academic editor and reviewer(s). This letter should be uploaded as separate file and labeled 'Response to Reviewers'.
- A marked-up copy of your manuscript that highlights changes made to the original version. This file should be uploaded as separate file and labeled 'Revised Manuscript with Track Changes'.
- An unmarked version of your revised paper without tracked changes. This file should be uploaded as separate file and labeled 'Manuscript'.

Please note while forming your response, if your article is accepted, you may have the opportunity to make the peer review history publicly available. The record will include editor decision letters (with reviews) and your responses to reviewer comments. If eligible, we will contact you to opt in or out.

We look forward to receiving your revised manuscript.

Kind regards,

Florian B. Mayr
Academic Editor
PLOS ONE


[Note: HTML markup is below. Please do not edit.]

Reviewers' comments:

Reviewer's Responses to Questions

**Comments to the Author**

1. If the authors have adequately addressed your comments raised in a previous round of review and you feel that this manuscript is now acceptable for publication, you may indicate that here to bypass the “Comments to the Author” section, enter your conflict of interest statement in the “Confidential to Editor” section, and submit your "Accept" recommendation.

Reviewer #1: All comments have been addressed

2. Is the manuscript technically sound, and do the data support the conclusions?

The manuscript must describe a technically sound piece of scientific research with data that supports the conclusions. Experiments must have been conducted rigorously, with appropriate controls, replication, and sample sizes. The conclusions must be drawn appropriately based on the data presented.

Reviewer #1: Yes

3. Has the statistical analysis been performed appropriately and rigorously?

Reviewer #1: Yes

4. Have the authors made all data underlying the findings in their manuscript fully available?

The [PLOS Data policy](http://www.plosone.org/static/policies.action#sharing) requires authors to make all data underlying the findings described in their manuscript fully available without restriction, with rare exception (please refer to the Data Availability Statement in the manuscript PDF file). The data should be provided as part of the manuscript or its supporting information, or deposited to a public repository. For example, in addition to summary statistics, the data points behind means, medians and variance measures should be available. If there are restrictions on publicly sharing data—e.g. participant privacy or use of data from a third party—those must be specified.

Reviewer #1: Yes

5. Is the manuscript presented in an intelligible fashion and written in standard English?

PLOS ONE does not copyedit accepted manuscripts, so the language in submitted articles must be clear, correct, and unambiguous. Any typographical or grammatical errors should be corrected at revision, so please note any specific errors here.

Reviewer #1: Yes

6. Review Comments to the Author

Please use the space provided to explain your answers to the questions above. You may also include additional comments for the author, including concerns about dual publication, research ethics, or publication ethics. (Please upload your review as an attachment if it exceeds 20,000 characters)

Reviewer #1: Chou et al. have significantly improved the methodology of this study. The interpretation of results is sound with expanded limitations and discussion. I agree with publication of these important results with minor revisions listed below.
Minor revisions:
1) Line 151-152: This sentence is confusing. Please re-word. “Male patients tend to be more likely to sepsis in all subperiods.”

**Author Reply:**

Thanks for your comment. We have re-worded accordingly. “There are more male patients than female in all subperiods.” (Line 151-152 in Section of ***Results***)

2) Lines 182-189: You use Odds Ratios, but in the methods section lines 133-134 you mention that odds ratios are biased measures given the prevalence of sepsis and therefore do not reliably predict risk. Please clarify.

**Author Reply:**

Thanks for your comment. To evaluate the impact of individual site of infection on the survival of sepsis patients, we fit a multivariable logistic regression model adjusting for age, sex, and comorbidity measures. We used the entire study period for this regression analysis to ensure adequate power to make reliable estimates of risk. Because the mortality rate for patients with sepsis is higher than 10% in this analysis, the rare disease assumption does not hold. As a result, risk ratios cannot be estimated by odds ratios. Therefore, we used the formula proposed by Zhang and Yu to approximate the relative risk. (1) We have corrected our manuscript, tables and figures accordingly.

“As a result, risk ratios cannot be estimated by odds ratios.” (Line 134 in Section of ***Methods***)

“Figure 4 shows the adjusted relative risk with 95% confidence intervals of infection site on the outcome of sepsis. Using primary bacteremia as reference, sepsis patients with intra-abdominal infection had the highest mortality (RR:4.21), followed by lower respiratory tract infection (RR: 3.84), biliary tract infection (RR: 3.24), systemic fungal infection (RR: 2.77), skin and skin structure infection (RR: 2.29), musculoskeletal infection (RR: 2.27), genitourinary traction infection (RR:2.19), or catheter related bloodstream infection (RR:2.15). Sensitivity analysis with Angus criteria showed similar trend of sepsis as our main results (Table S4-7 and Fig S1-3)

Fig 4. Survival impact of individual infection site in relation to primary bacteremia. The risk estimates were adjusted for all covariates listed in supporting table 1. RR refers to the relative risk. LCL and UCL refer to lower and upper confidence limits, respectively.” (Line 182-191 in Section of ***Results***)

“Supporting Figure 3. Sensitivity Test - Survival impact of individual infection site in relation to primary bacteremia. The risk estimates were adjusted for all covariates listed in Supporting table 7. RR refers to the relative risk. LCL and UCL refer to lower and upper confidence limits, respectively.

Supporting Table 1. Covariates with associated relative risk in the outcome regression model.” (Line 371-376 in Section of ***Supporting Information***)

“Supporting Table 7. Sensitivity Test - Covariates with associated relative risk in the outcome regression model.” (Line 386-388 in Section of ***Supporting Information***)

3) Lines 209-210: “One example being the surviving sepsis campaign being started at the beginning of this study who could account for increased incidence of sepsis”. This is confusing. Please re-word.

**Author Reply:**

Thanks for your comment. We have re-worded accordingly.

“For example, the surviving sepsis campaign started at the beginning of this study could account for early sepsis recognition and decreasing mortality with early antibiotic administration and three-hour bundle therapy (20-22).” (Line 208-210 in Section of ***Discussions***)

4) Lines 214-215: “which is common etiologic agent of pneumonia.” Please fix grammar.

**Author Reply:**

Thanks for your comment. We have re-worded accordingly.

“Currently preventative strategies mostly aimed at *streptococcus pneumoniae*, which is the common pathogen of pneumonia.” (Line 212-213 in Section of ***Discussions***)

7. PLOS authors have the option to publish the peer review history of their article ([what does this mean?](https://journals.plos.org/plosone/s/editorial-and-peer-review-process#loc-peer-review-history)). If published, this will include your full peer review and any attached files.


If you choose “no”, your identity will remain anonymous but your review may still be made public.


**Do you want your identity to be public for this peer review?** For information about this choice, including consent withdrawal, please see our [Privacy Policy](https://www.plos.org/privacy-policy).

Reviewer #1: Yes: Matthew K Hensley


[NOTE: If reviewer comments were submitted as an attachment file, they will be attached to this email and accessible via the submission site. Please log into your account, locate the manuscript record, and check for the action link "View Attachments". If this link does not appear, there are no attachment files to be viewed.]


While revising your submission, please upload your figure files to the Preflight Analysis and Conversion Engine (PACE) digital diagnostic tool, <https://pacev2.apexcovantage.com/>. PACE helps ensure that figures meet PLOS requirements. To use PACE, you must first register as a user. Registration is free. Then, login and navigate to the UPLOAD tab, where you will find detailed instructions on how to use the tool. If you encounter any issues or have any questions when using PACE, please email us at [figures@plos.org](mailto:figures@plos.org). Please note that Supporting Information files do not need this step.

Reference:

1. Zhang J, Yu KF. What's the relative risk? A method of correcting the odds ratio in cohort studies of common outcomes. *Jama* 1998; 280: 1690-1691.
